# Thalamus Radiomics-Based Disease Identification and Prediction of Early Treatment Response for Schizophrenia

**DOI:** 10.3389/fnins.2021.682777

**Published:** 2021-07-05

**Authors:** Long-Biao Cui, Ya-Juan Zhang, Hong-Liang Lu, Lin Liu, Hai-Jun Zhang, Yu-Fei Fu, Xu-Sha Wu, Yong-Qiang Xu, Xiao-Sa Li, Yu-Ting Qiao, Wei Qin, Hong Yin, Feng Cao

**Affiliations:** ^1^The Second Medical Center, Chinese PLA General Hospital, Beijing, China; ^2^Department of Clinical Psychology, Fourth Military Medical University, Xi’an, China; ^3^Military Medical Psychology School, Fourth Military Medical University, Xi’an, China; ^4^School of Life Sciences and Technology, Xidian University, Xi’an, China; ^5^Peking University Sixth Hospital/Institute of Mental Health and Key Laboratory of Mental Health, Peking University, Beijing, China; ^6^Department of Clinical Aerospace Medicine, School of Aerospace Medicine, Fourth Military Medical University, Xi’an, China; ^7^Department of Radiology, Xijing Hospital, Fourth Military Medical University, Xi’an, China; ^8^Department of Psychiatry, Xijing Hospital, Fourth Military Medical University, Xi’an, China

**Keywords:** schizophrenia, thalamus, radiomics, machine learning, diagnosis, treatment

## Abstract

**Background:**

Emerging evidence suggests structural and functional disruptions of the thalamus in schizophrenia, but whether thalamus abnormalities are able to be used for disease identification and prediction of early treatment response in schizophrenia remains to be determined. This study aims at developing and validating a method of disease identification and prediction of treatment response by multi-dimensional thalamic features derived from magnetic resonance imaging in schizophrenia patients using radiomics approaches.

**Methods:**

A total of 390 subjects, including patients with schizophrenia and healthy controls, participated in this study, among which 109 out of 191 patients had clinical characteristics of early outcome (61 responders and 48 non-responders). Thalamus-based radiomics features were extracted and selected. The diagnostic and predictive capacity of multi-dimensional thalamic features was evaluated using radiomics approach.

**Results:**

Using radiomics features, the classifier accurately discriminated patients from healthy controls, with an accuracy of 68%. The features were further confirmed in prediction and random forest of treatment response, with an accuracy of 75%.

**Conclusion:**

Our study demonstrates a radiomics approach by multiple thalamic features to identify schizophrenia and predict early treatment response. Thalamus-based classification could be promising to apply in schizophrenia definition and treatment selection.

## Introduction

Driven by the need for precision medicine, a quest for accurate diagnosis and treatment was recently noted in the management of schizophrenia. A variety of abnormalities in the thalamus is associated with this disorder, including reduced volume (Pergola et al., 2015; Brugger and Howes, 2017; Dietsche et al., 2017; Dorph-Petersen and Lewis, 2017) and disrupted structural and functional connections to the cortices (Pergola et al., 2015; Giraldo-Chica and Woodward, 2017; Murray and Anticevic, 2017), as well as increased perfusion (Scheef et al., 2010; Zhu et al., 2015) and weaker correlation between glucose metabolism and dopaminergic state (Mitelman et al., 2019). Copious neuroimaging studies suggest thalamic association with schizophrenia, ranging from region to network level.

Task-state studies have found increased blood oxygenation level-dependent response to retrieval in the thalamus among schizophrenia patients (Stolz et al., 2012). Meanwhile, resting-state functional connectivity studies have reported thalamic abnormal connectivity with the bilateral cerebellum, anterior cingulate cortex, and multiple sensory-motor regions (Ferri et al., 2018). Effective connectivity by means of dynamic causal modeling revealed a deficit sensitivity of auditory cortex to its thalamic afferents in schizophrenia (Li et al., 2017). In addition, disrupted coactivation within resting-state networks analysis has been observed in the thalamus (Cui et al., 2017a). Both functional and structural imaging findings support dysconnectivity of the thalamus and cerebellum (Liu et al., 2011). As the neuroanatomical and neurochemical theories implicated in the pathophysiology of schizophrenia, the notion of emphasizing psychopathological processes mediated by the thalamus (Parnaudeau et al., 2018) should also be paralleled by identifying patients and predicting treatment response *via* multi-dimensional thalamic features.

A number of studies indicate that magnetic resonance imaging (MRI) techniques have provided insights into the classification and prediction in schizophrenia. MRI combined with machine learning technique represents a promising approach to distinguish patients with schizophrenia from healthy population, and responders from non-responders (de Filippis et al., 2019; Wang et al., 2019). In general, previous studies have related to the classification of schizophrenia using resting-state functional MRI (Anderson et al., 2010; Shen et al., 2010; Anderson and Cohen, 2013; Skatun et al., 2017; Cui et al., 2018; Huang et al., 2019; Zeng et al., 2018), structural MRI (Liang et al., 2018; Mikolas et al., 2018; Cui et al., 2019b; Liu et al., 2020), or their combination (Cui et al., 2021a). More importantly, classification approaches are able to aid subtyping symptoms of schizophrenia (Dwyer et al., 2018) and *trans-*diagnostic discrimination between schizophrenia and bipolar disorder (Arribas et al., 2010; Schnack et al., 2014; Rashid et al., 2016). In particular, MRI may be able to predict the response of treatments in schizophrenia, including structural (Fung et al., 2014; Hutcheson et al., 2014; Molina et al., 2014; Morch-Johnsen et al., 2015; Premkumar et al., 2015; Altamura et al., 2017; Dusi et al., 2017; Francis et al., 2018) and functional (Hadley et al., 2014; Kraguljac et al., 2016a,b; Sarpal et al., 2016; Doucet et al., 2018; Shafritz et al., 2018; Cui et al., 2019a) MRI (see Cui et al., for review; Cui et al., 2019a). These studies involved MRI features such as gray matter or white matter volume, cortical thickness, morphology of gyrus, and brain activation and connectivity with time of outcome assessment arranging from 6 weeks to 3 years. The structural MRI findings have shown a linkage between clinical improvements and higher gray matter volume [e.g., bilateral caudate (Hutcheson et al., 2014), bilateral lentiform and striatum (Fung et al., 2014), orbitofrontal cortex (Premkumar et al., 2015), and total brain (Altamura et al., 2017)], thinner right prefrontal (Molina et al., 2014) and thicker left caudal middle frontal cortical thickness (Francis et al., 2018), and rightward orbitofrontal cortex (Premkumar et al., 2015). In contrast, poor response has been linked to thinner left orbitofrontal cortex and left anterior cingulate cortex (Morch-Johnsen et al., 2015), and decreased right dorsolateral prefrontal cortex white matter volume (Dusi et al., 2017). Several functional MRI studies have reported greater activation in the anterior cingulate cortex in a simple response conflict task (Shafritz et al., 2018), and increased regional activity in the left postcentral gyrus/inferior parietal lobule (Cui et al., 2019a) and distinctive striatal functional connectivity (Sarpal et al., 2016) for responders. Hippocampal connectivity (Kraguljac et al., 2016b), connectivity within the dorsal attention network (Kraguljac et al., 2016a), and connectivity between ventral tegmental area/midbrain and the dorsal anterior cingulate cortex (Hadley et al., 2014) have been found to positively correlate to changes in symptoms. However, these potential predictors are inordinately heterogeneous and, to our knowledge, much earlier prediction of treatment response has not been identified.

Emerging evidence suggests structural and functional disruptions of the thalamus in schizophrenia, but whether thalamus abnormalities are able to be used for classification and prediction in schizophrenia remains to be determined. Thalamic features with successful level prediction of electroconvulsive therapy (ECT) response have been identified by radiomics (Xi et al., 2020). An opinion article in this journal illustrates the application of MRI and radiomics/machine learning methods to the study of schizophrenia (see Cui et al. for review; Cui et al., 2021b). Therefore, we aimed to validate a method of classification for schizophrenia and prediction of treatment response by multi-dimensional thalamic features derived from structural MRI using radiomics approaches. Relying on the thalamic association with schizophrenia, we hypothesized that thalamus-based classification and prediction could play a role in individualized diagnosis and treatment of schizophrenia as an objective and useful tool in this study.

## Materials and Methods

This study was approved by the Institutional Ethics Committee, First Affiliated Hospital (Xijing Hospital) of the Fourth Military Medical University. All participants (or their parents for those under age of 18 years) gave written informed consent after a full description of the aims and design of the study. Table 1 provides further details on the two patient and control populations.

**TABLE 1:** Clinical and demographical data.

**Characteristics**	**Patients (*n* = 191)**	**Healthy controls (*n* = 199)**	***P*-values**	**Responders (*n* = 61)**	**Non-responders (*n* = 48)**	***P*-values**
Age (years)	25 ± 7	29 ± 9	<0.001	24 ± 6	27 ± 8	0.036
Gender (M/F)	107/84	109/90	0.804	40/21	26/22	0.226
Education level (years)	12 ± 3	14 ± 4	<0.001	12 ± 2	13 ± 3	0.579
Duration of illness (months)	19 ± 26	–	–	17 ± 21	21 ± 31	0.368
**PANSS score at baseline**						
Total score	90 ± 17	–	–	90 ± 20	89 ± 14	0.774
Positive score	23 ± 6	–	–	23 ± 7	23 ± 7	0.847
Negative score	21 ± 8	–	–	21 ± 8	22 ± 8	0.548
General score	46 ± 9	–	–	46 ± 10	45 ± 7	0.329
**PANSS score at discharging**						
Total score	–	–	–	60 ± 15	80 ± 12	<0.001
Positive score	–	–	–	14 ± 5	20 ± 5	<0.001
Negative score	–	–	–	14 ± 6	20 ± 7	<0.001
General score	–	–	–	32 ± 8	40 ± 6	<0.001
Changes in PANSS score (%)	–	–	–	51 ± 16	16 ± 11	<0.001
Stay in hospital (days)	–	–	–	17 ± 5	15 ± 5	0.115
Antipsychotic dose (mg/day)^a^	–	–	–	10 ± 4	10 ± 4	0.388

*Data are means ± standard deviations.*

*^*a*^Defined Daily Dose (DDD).*

### Participants

The inclusion and exclusion criteria are shown in previous studies (Cui et al., 2018, 2019a,2019b). The first dataset included 100 patients with schizophrenia patients and 92 healthy controls. The structural clinical interview for Diagnostic and Statistical Manual of Mental Disorders, Fourth Edition, Text Revision (DSM-IV-TR) was used, and consensus diagnoses were made using all the available information. The second dataset included 91 patients and 107 healthy controls, and DSM, Fifth Edition (DSM-5) was used. Each patient was assessed by using the Positive and Negative Syndrome Scale (PANSS) at the time of imaging (Cui et al., 2018, 2019a,2019b).

Data were collected from May 2011 to December 2013 (dataset 1) and from April 2015 to December 2017 (dataset 2) in the Department of Psychiatry, Xijing Hospital, respectively, including inpatients undergoing their first or single hospitalization and outpatients seeking help. Inclusion criteria for patients are as follows: (1) they were assessed by two senior clinical psychiatrists, and consensus diagnosis of schizophrenia was made; (2) PANSS score was not less than 60 at the time of imaging; (3) all subjects were right handed, and their biological parents were of the Han Chinese ethnic group. Two groups of healthy controls without any reported psychotic syndrome (as assessed by psychiatrists) were recruited by advertisement from the local community.

Exclusion criteria for patients included the following: (1) presence of another psychiatric disorder; (2) history of repetitive transcranial magnetic or current stimulation, or a history of behavioral treatment; (3) history of clinically significant neurological, neurosurgical, or medical illnesses; (4) substance abuse within the prior 30 days or substance dependence within the prior 6 months; and (5) pregnancy or any other MRI contraindications, e.g., cardiac pacemakers and other metallic implants. Exclusion criteria for healthy controls included the following: (1) presence of any psychotic syndrome; (2) history of receiving antipsychotics, repetitive transcranial magnetic stimulation, transcranial current stimulation, or behavioral treatment; (3) history of clinically significant neurological, neurosurgical, or medical illnesses; (4) substance abuse within the prior 30 days or substance dependence within the prior 6 months; and (5) pregnancy or MRI contraindications, e.g., cardiac pacemakers and other metallic implants.

A total of 109 patients (67 from the first dataset; 42 from the second dataset) had clinical data of early treatment response. The majority of patients received second-generation antipsychotics, and the minority of patients received first-generation antipsychotics. Treatment response at discharging was assessed using percentage change of PANSS score: PANSS percentage change = (total score_1_ − total score_0_) × 100 ÷ (total score_0_ − 30). Responders were defined as 30% reduction in PANSS total scores previously used (Cui et al., 2019a).

### Image Acquisition

**FIGURE 1:**
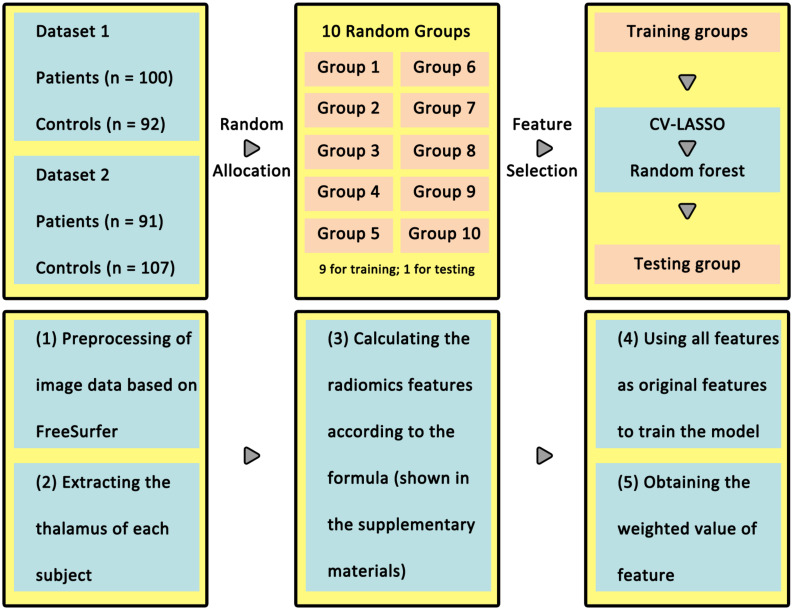
Workflow for analysis in classification of patients and healthy controls. In the **upper panel**, all of the participants were randomly divided into 10 groups, nine for training and one for testing. The **lower panel** summarizes radiomics steps. The radiomics features were extracted using CV-LASSO in the training group and validated in the testing group using random forest.

High-resolution structural imaging was acquired on a Siemens 3.0 T Magnetom Trio Tim MR scanner (the first dataset) or General Electric (GE) Discovery MR750 3.0 T scanner (the second dataset) using protocols described elsewhere (Xi et al., 2016; Cui et al., 2017a,b). All the imaging data were collected in the Department of Radiology, Xijing Hospital. A custom-built head coil cushion and earplugs were used to minimize head motion and dampen scanner noise. During data acquisition, subjects were asked to remain alert with eyes closed and keep their head still. Participants in dataset 1 underwent scanning using a 3.0-T Siemens Magnetom Trio Tim scanner and an eight-channel phased array head coil (Siemens, Germany). Participants in dataset 2 underwent scans on a GE Discovery MR750 3.0-T scanner and an eight-channel phased array head coil (Milwaukee, WI, United States). Detailed parameters of high-resolution T1-weighted anatomical data are listed in Table 2. As performed previously (Cui et al., 2018), steps for the following analysis are shown in Figure 1.

**TABLE 2:** Scanning parameters of T1-weighted imaging.

	**The first dataset**	**The second dataset**
Scanner	Siemens	GE
TR (ms)	2530	8.2
TE (ms)	3.5	3.2
Flip angle (°)	7	12
FOV (mm^2^)	256 × 256	256 × 256
Matrix	256 × 256	256 × 256
Slice thickness (mm)	1	1
Section gap (mm)	0	0
Number of slices	192	196

### Imaging Data Preprocessing and Extracting Thalamus

T1-weighted image processing was performed using the FreeSurfer image analysis suite (version 6.0.0)^1^. Data preprocessing was to register the original high-resolution structural image of each subject to standard template, and project it back to each subject to extract thalamus tissue. The preprocessing process is the standard process of the FreeSurfer toolkit.

Briefly, preprocessing was performed with the following steps: (i) skull stripping, (ii) normalization to a standard anatomical template (Talairach and Tournoux, 1988), (iii) correction for bias-field inhomogeneity, (iv) segmentation of subcortical white matter and deep gray matter volumetric structures (Fischl et al., 2002, 2004), (v) gray–white matter boundary tessellation and a series of deformation procedures that consist of surface inflation (Dale et al., 1999), and (vi) registration to a spherical atlas (Fischl et al., 1999) and parcellation of the cerebral cortex into units based on the gyral and sulcal structures (Fischl et al., 2004). In line with previous studies using the radiomics features from the bilateral structures in mental disorders (Chaddad et al., 2017; Park et al., 2020), we considered the bilateral thalami as regions of interest. In addition, the workflow of extracting thalamus was as follows: (i) the T1 images after preprocessing were matched to Anatomical Automatic Labeling (AAL) cortical and subcortical 1 mm × 1 mm × 1 mm atlas, and got the transformation matrix; (ii) use the inverse matrix of the transformation matrix to register AAL to individual space. After preprocessing, each subject’s thalamus was registered to the standard space with consistent resolution. In this study, we did not perform interpolation in image processing^2^.

### Radiomics Features

The following analysis is based on the guidelines in radiomics (Lambin et al., 2017; Vallieres et al., 2018). Each image feature calculation formula is provided in the Supplementary Material, and they were based on the image biomarker standardization initiative^3^. Four types of radiomics features were used to quantify thalamic characteristics (Aerts et al., 2014): (i) first-order features, (ii) second-order features, (iii) texture features, and (iv) wavelet features, which have been used in previous studies (Gong et al., 2020; Xi et al., 2020). The first group quantified thalamus intensity characteristics using first-order statistics, calculated from the histogram of all thalamus voxel intensity values (14 radiomic features: energy, entropy, kurtosis, maximum, mean, mean absolute deviation, median, minimum, range, root mean square, skewness, standard deviation, uniformity, and variance). Group 2 consists of features based on the shape of the thalamus (eight radiomics features: compactness 1 and 2, maximum 3D diameter, spherical disproportion, sphericity, surface area, surface-to-volume ratio, and volume). Group 3 consists of textual features that are able to quantify intra-thalamus heterogeneity differences in the texture that is observable within the thalamus volume. These features are calculated in all three-dimensional directions within the thalamus volume, taking the spatial location of each voxel compared with the surrounding voxels into account. In this research, texture features describing patterns or the spatial distribution of voxel intensities were calculated from, respectively, gray level co-occurrence (GLCM) and gray level run-length (GLRLM) texture matrices. Texture matrices were determined considering 26 connected voxels. Group 4 wavelet transform effectively decouples textural information by decomposing the original image in low and high frequencies. Here, the first- and second-order features and textural features of eight directions (the original images were decomposed into eight directions) were calculated. All feature algorithms were implemented in Matlab 2016a (MathWorks, Natick, MA, United States). In the process of feature extraction, we performed the discretization and used 2 mm × 2 mm × 2 mm as voxels to extract the imaging features of the thalamus and take the mean value.

### Feature Selection, Classification Model, and Efficacy Prediction

Ten-fold cross-validation (CV) was used to assess the reliability of the classification model (Figure 1). Briefly, 390 subjects (190 patients) were randomly separated into 10 groups. Each time, one group in turn was used as a test group and the other nine groups were used as training group.

A total of 4019 radiomics features were selected as initial features. After that, we used a 10-fold CV-based Least Absolute Shrinkage and Selection Operator (CV-LASSO) method to further select features. Briefly, subjects in the training group were again randomly separated into 10 groups. Each time, one group in turn was excluded from the dataset, and the LASSO (Sauerbrei et al., 2007) method with mean of square error (MSE) as the cost function was used on the remaining nine groups to narrow down the initial features into the most important features according to the MSE + 1SE criteria (Sauerbrei et al., 2007). This step was repeated 10 times, which resulted in 10 different groups of selected features. Finally, the edges that were included in the selected feature group at least N times (i.e., occurring N times) were selected as LASSO features for further analysis. Next, the random forest (RF) method was used to construct the classification model based on LASSO features in training group. The accuracy, sensitivity, specificity, and recall indices of the constructed model were calculated using testing group. Considering any confound factors due to data from two scanners, the differences of features selected between participants in the two datasets were compared.

All these steps above were repeated 10 times. As for the setting of P0, N, and the number of trees t in RF, we used grid-search method to find them. These parameters were set at a group of specific values when the accuracy index of the constructed classification model achieved the maximum. The P0 was set from 0.01 to 0.1 with a step of 0.01. The N was set from 1 to 10 with a step of 1. The t was set from 5 to 100 with a step of 5.

To avoid the random group effect, we repeated the 10-fold CV 100 times. For each time, a new random group was split. The mean ± standard deviation of each index across the 1000 testing groups (10 × 100) was used to assess the performance and stability of the constructed model. Finally, 1000 times permutation test (group label permutation) was performed to check if our results were significantly different from random labels.

### Validation

Finally, we used another machine learning method, support vector machine, to estimate the status of each participant (schizophrenia or control; responder or non-responder) *via* intra- and inter-dataset CV (Cui et al., 2018).

## Results

### Clinical Characteristics

Table 1 shows the full description of demographic and clinical characteristics of patients and healthy controls. No significant difference was found in gender between patients and healthy controls. For patients, there was statistical difference in being younger (*P* < 0.001) and having a lower education level (*P* < 0.001).

### Feature Selection

**FIGURE 2:**
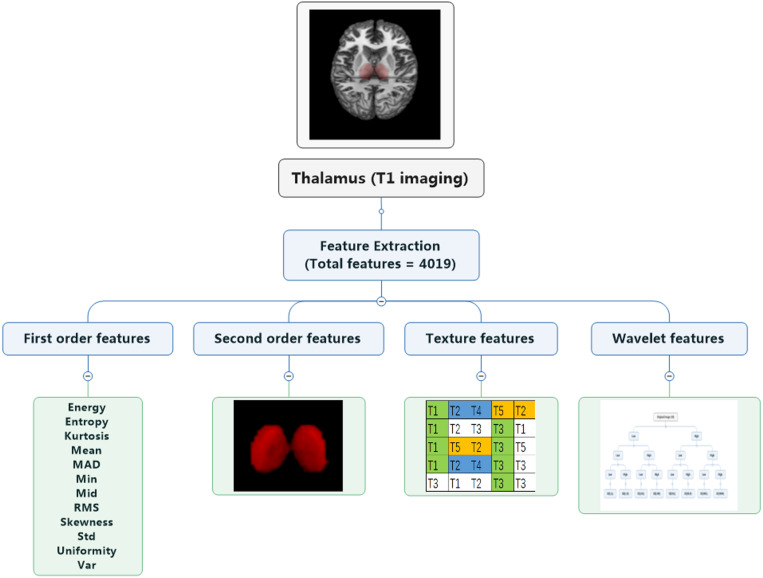
Extraction of radiomics features. Four groups of radiomics features include first-order features, second-order features, texture features, and wavelet features. A total of 4019 features were extracted.

The RF was performed for the high-resolution T1-weighted imaging. In this study, 4019 radiomics features were extracted (Figure 2 and see the Supplementary Material), resulting in 12 features for identifying patients (“W1.Mid,” “W1.SRE_8,” “W2.LRHGLE_8,” “W3.Min,” “W4.Co_Corr_12,” “W4.Co_Var_13,” “W5.Co_Corr_11,” “W5.RLN_9,” “W6.Co_Corr_2,” “W6.Co_Corr_7,” “W7.IMC1_9,” and “W9.Co_Corr_12”) and four features for predicting treatment response (“W1.LRE_9,” “W3.Min,” “W6.Co_Corr_7,” and “W6.Co_Var_7”). For the selected features, we performed comparison between subjects on two scanners, e.g., patients/healthy controls on Siemens scanner and GE scanner, and no significant difference was found between two scanners by *t*-tests.

### Classification Performance

**FIGURE 3:**
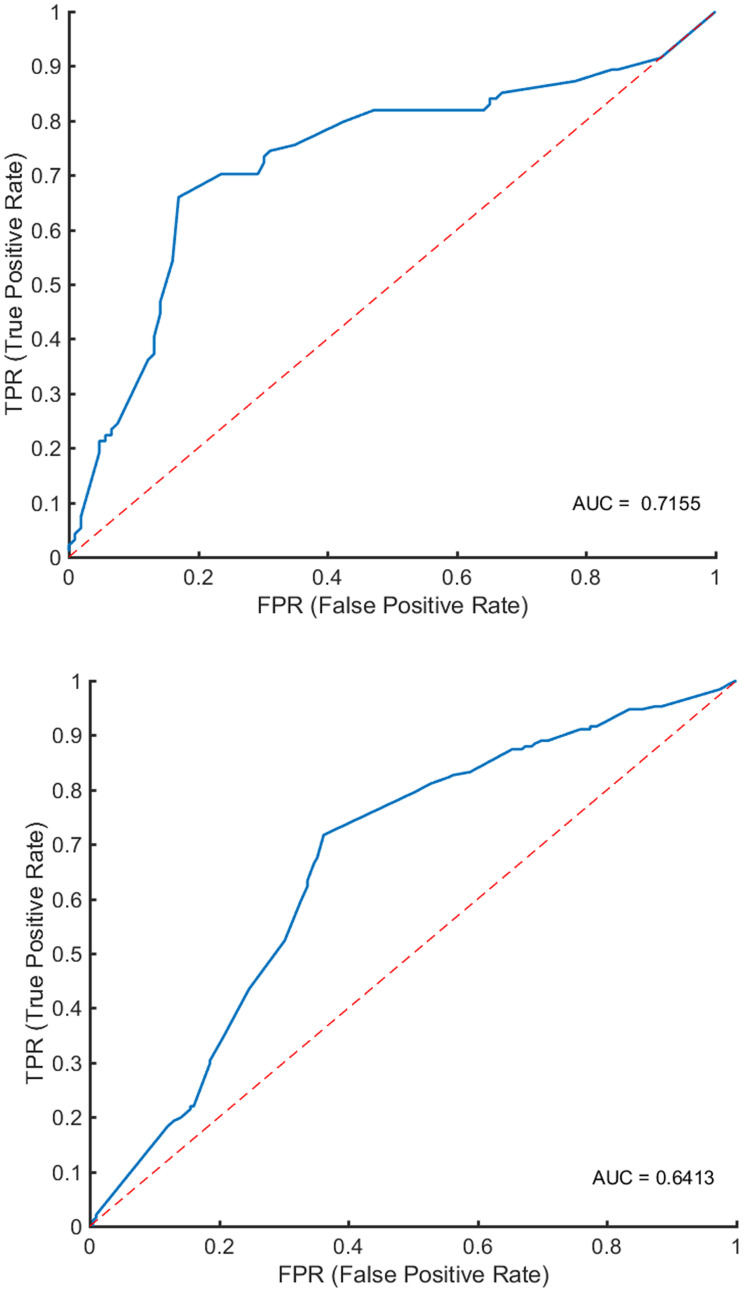
Classification performance. In the **upper panel**, ROC analyses showed an AUC of 0.7155 for predicting early treatment response. In the **lower panel**, ROC analyses showed an AUC of 0.6413 for identifying patients with schizophrenia.

Figure 3 and Table 3 show the classification performance. Using 12 features, the RF classifier accurately discriminated patients from healthy controls on the basis of the receiver operating characteristic (ROC) curve, with an accuracy of 68%. Four features were further confirmed in the prediction of treatment response, with an accuracy of 75%. The DeLong test suggested that the model of the area under curve (AUC) of the ROC analysis for response prediction was superior to that for diagnosis (*P* = 0.015).

**TABLE 3:** Classification performance.

	**Accuracy**	**Sensitivity**	**Specificity**	**AUC**	**Features**
Diagnosis (191 patients and 199 controls)	0.68 ± 0.04	0.60 ± 0.31	0.61 ± 0.30	0.64 ± 0.23	“W1.Mid”; “W1.SRE_8”; “W2.LRHGLE_8”; “W3.Min”; “W4.Co_Corr_12”; “W4.Co_Var_13”; “W5.Co_Corr_11”; “W5.RLN_9”; “W6.Co_Corr_2”; “W6.Co_Corr_7”; “W7.IMC1_9”; “W9.Co_Corr_12”
Prediction (61 responders and 48 non-responders)	0.75 ± 0.08	0.65 ± 0.25	0.80 ± 0.23	0.72 ± 0.12	“W1.LRE_9”; “W3.Min”; “W6.Co_Corr_7”; “W6.Co_Var_7”

*AUC, area under the curve; “W1”–“W9”, Wavelet features; “_1”–“_13”, direction of each feature; Mid, middle; SRE, short run emphasis; LRHGLE, long-run high gray-level emphasis; Min, minimum; Co_Corr, correlation; RLN, run length non-uniformity; IMC, informational measure of correlation; LRE, long-run emphasis; Var, variance.*

### Validation

Combining radiomics and support vector machine method, thalamic features had an accuracy arranging from 63 to 71% for classification with intra- and inter-dataset CVs (Table 4).

**TABLE 4:** Classification performance using intra- and inter-dataset cross-validation.

	**Accuracy**	**Sensitivity**	**Specificity**	**Features**
**Intra-dataset cross-validation (80% dataset 1 and dataset 2 for training and the other 20% for testing)**				
Diagnosis (17 features)	68.37%	71.15%	70.62%	W1.Mid; W1.Min; W1.Mid; W2.RMS; W2.Surface; W2.SVR; W2.Volume; W2.SRE_8; W2.Homo2_13; W3.Min; W4.Co_Corr_12; W4.Co_Var_13; W5.Co_Corr_11; W5.RLN_9; W6.Co_Corr_2; W6.Co_Corr_7; W7.IMC1_9
Prediction (7 features)	71.01%	72.53%	71.69%	W1.SRLGLE_1; W1.Compactness1; W2.Energy; W2.MAD; W3.Min; W6.Cluster_Shade_mean; W8.Cluster_Shade_8
**Inter-dataset cross-validation (dataset 1 training, dataset 2 testing)**				
Diagnosis (12 features)	65.19%	63.21%	68.55%	W1.Mid; W1.SRE_8; W1.Min; W2.RMS; W2.Surface; W2.Homo2_13; W3.Min; W4.Co_Corr_12; W5.Co_Corr_11; W5.RLN_9; W6.Co_Corr_2; W9.SRHGLE_5
Prediction (5 features)	68.36%	65.75%	69.73%	W1.LRE_9; W1.HGLRE_3; W2.Energy_GLCM_3; W7.Max_GLCM_1; W8.AutoCorr_2
**Inter-dataset cross-validation (dataset 2 training, dataset 1 testing)**				
Diagnosis (10 features)	63.88%	67.56%	66.46%	W1.LGLRE_11; W1.SRE_6; W2.Sum_var_mean; W2.SRLGLE_6; W4.Dissimilarity_1; W5.Dissimilarity_mean; W5.SRLGLE_4; W7.Diff_entropy_13; W7.Homo2_13; W9.IMC1_mean
Prediction (4 features)	65.21%	69.02%	64.35%	W1.Min; W2.Uniformity; W8.Energy_GLCM_1; W9.LRHGLE_mean

*“W1”–“W9”, Wavelet features; “_1”–“_13”, direction of each feature; AutoCorr, autocorrelation; Co_Corr, correlation; GLCM, gray-level co-occurrence matrix; Homo, homogeneity; IMC, informational measure of correlation; LGLRE, low gray-level run emphasis; LRE, long-run emphasis; LRHGLE, long-run high gray-level emphasis; MAD, median absolute deviation; Max, maximum; Mid, middle; Min, minimum; RLN, run length non-uniformity; RMS, root mean square; SRE, short-run emphasis; SRHGLE, short-run high gray-level emphasis; SRLGLE, short-run low gray-level emphasis; Var, variance.*

## Discussion

Using radiomics approach and RF, we explored whether multi-dimensional thalamic features define patients with schizophrenia/patients who responded to treatment in this study, resulting in an accuracy of 68% for distinguishing patients with schizophrenia from healthy population and an accuracy of 75% for prediction of early treatment response. Furthermore, support vector machine method revealed similar results through intra- and inter-dataset CV. Our findings might help to facilitate objective diagnosis and treatment selection based on quantitative and specific thalamic signature, reflecting its pathophysiology underlying schizophrenia (Tomaszewski and Gillies, 2021).

With the exception of showing conventional features of the thalamus, we also provide newly developed high-throughput features on structural imaging. Findings from imaging and postmortem studies of whole thalamus volume and other structural measures are mixed in schizophrenia and may be influenced by methods, disease state, and the fact that the thalamus is an exceptionally heterogeneous structure. Convergent findings based on multimodality MRI provide support for these neural substrates mediated by the thalamus in schizophrenia (Huang et al., 2015; Xi et al., 2016), suggesting that thalamic abnormalities are implicated in the pathophysiology of this mental disorder. Detecting schizophrenia based on functional connectome is driven by a distributed bilateral network including the thalamus and temporal regions (Lei et al., 2019). As for predicting treatment response, higher baseline glutamate/creatine in the thalamus was seen in non-responders on aripiprazole monotherapy at week 6 and on naturalistic antipsychotic treatment at week 26 compared with healthy controls (Bojesen et al., 2019). Extending previous findings, this evidence is the fundamental basis for disease definition and treatment selection by means of thalamic features using radiomics approach.

Neuroimaging findings have not been used for psychotic disorders clinically, because they are “not sufficiently sensitive or specific for reliable diagnosis in individual patients” (Lieberman and First, 2018). Therefore, from the perspective of methodology, *via* the quickly developed radiomics strategy, the diagnostic performance of multi-dimensional thalamic features is proved liable for identifying individual patients with schizophrenia and predicting early treatment response. The accuracy varied from 82.1 to 87.09% in two previous similar studies by radiomic features from the bilateral hippocampal subfields (Park et al., 2020) and whole brain functional connectivity (Cui et al., 2018). We obtained an accuracy of 68% using the high-throughput thalamic features, in comparison to the diagnostic performance with an accuracy of 78.3% by resting-state networks features (Skatun et al., 2017) and 73.0–81.3% by resting-state connectivity (Mikolas et al., 2016; Huang et al., 2019). Radiomics is considered as the bridge between medical imaging and personalized medicine, and promising to play a central role in the context of psychiatry.

For the cutoff of less than 25% PANSS/Brief Psychiatric Rating Scale (BPRS) reduction, the overall non-response is 43%, and for the cutoff of less than 50% reduction, it is 66.5% (Samara et al., 2019). In line with the finding from randomized controlled trials that the response was assessed at 4–6 weeks, 48 out of 109 (44%) were non-responders for less than 30% PANSS reduction assessed at 2–3 weeks (15–17 days) in this study. Moreover, the olanzapine equivalent was 10 ± 4 mg/day for both responders and non-responders. Our results demonstrate a radiomics approach by multiple thalamic features to predict early treatment response with an accuracy of 75%, an increased level relative to diagnosis. In addition to MRI, genetic evidence indicates schizophrenia polygenic risk score as a predictor of response to antipsychotics in patients with first-episode psychosis (Zhang et al., 2019). An analysis combining neuroimaging and genetics is needed to facilitate the prediction of antipsychotic efficacy in the future. Moreover, radiomics risk modeling combined with time-to-event analysis will contribute to clarifying treatment response (Leger et al., 2017).

An issue of this study that needs to be pointed out is the absence of validation in an independent cohort. Validation could help to confirm the discriminating capacity from different scanners and sites with heterogeneity. A previous study combined independent data of KaSP (Karolinska Schizophrenia Project) and HUBIN (Human Brain Informatics) (Skatun et al., 2017), supporting generalizability across heterogeneous samples. Features across MRI scanners with no difference suggest the repeatability (Cui et al., 2018, 2021a). In the next step, the combination of data from different scanners could consolidate and promote the generalizability of MRI findings in clinical practice. As DSM-5 stated (American Psychiatric Association, 2013), “The peak age at onset for the first psychotic episode is in the early- to mid-20s for males and in the late-20s for females.” Our sample included patients with a wide age range, so potential confounders of brain development could not be excluded. A mixed group of high school students and young adults in the study reflects the clinical heterogeneity of schizophrenia. Besides, because of a very small number of patients with relatively long medical history and no precise boundary between short and long duration of illness, we were unable to perform meaningful subgroup analyses, which may introduce an effect on the results owing to the design of a naturalistic study. Finally, connectomics defined by MRI and genomics in neuropathology gain ground on brain disorder (Jiang et al., 2020); hence, *trans-*omics is promising to shape a refined diagnosis and prediction in schizophrenia. No “one fits all” omics approach exists in this field (Shiri et al., 2020). It depends on the study design.

Our study demonstrates a radiomics approach by multiple thalamic features to diagnose schizophrenia and predict early treatment response with a comparable accuracy. Combining novel machine learning models, radiomics studies try to break the boundary and tend to explore transdiagnostic characteristics of mental disorders (Cui et al., 2021b), transforming the guidance of diagnosis and treatment selection for mental disorders in the future.

## Data Availability Statement

The data analyzed in this study is subject to the following licenses/restrictions: There was no relevant provision concerning public access to data when participants were included, so the data in this study could not be publicly available. Requests to access these datasets should be directed to the corresponding author (L-BC).

## Ethics Statement

The studies involving human participants were reviewed and approved by the Institutional Ethics Committee, First Affiliated Hospital (Xijing Hospital) of the Fourth Military Medical University. Written informed consent to participate in this study was provided by the participants’ legal guardian/next of kin.

## Author Contributions

L-BC, HY, WQ, and FC: guarantors of integrity of entire study. L-BC, Y-JZ, H-LL, and LL: literature research and experimental studies. L-BC and LL: statistical analysis. All authors: study concepts/study design or data acquisition or data analysis/interpretation, manuscript drafting or manuscript revision for important intellectual content, approval of final version of submitted manuscript, clinical studies, and manuscript editing.

## Conflict of Interest

The authors declare that the research was conducted in the absence of any commercial or financial relationships that could be construed as a potential conflict of interest.

## Footnotes

1 http://surfer.nmr.mgh.harvard.edu/

2 https://ibsi.readthedocs.io/en/latest/

3 https://arxiv.org/abs/1612.07003
