# Supplementary material for: Thalamus Radiomics-Based Disease Identification and Prediction of Early Treatment Response for Schizophrenia
Source: Front Neurosci. 2021 Jul 5;15:682777. doi: 10.3389/fnins.2021.682777 (PMC8289251; doi:10.3389/fnins.2021.682777)
Supplement: Supplementary file 1 [file Data_Sheet_1.docx]

### First order feature

$R$: Three dimensional ROI matrix after segmentation, $i:$ Voxel strength value, $N_{v}$: the total number of voxels in ROI, $H$: First order gray statistical histogram of R, $N_{r}$: the number of discrete strength values in 𝑅 (𝑖) (Discrete Intensity Levels), $\bar{R}$: The mean value of R.

Energy:

$$\begin{aligned} energy=\sum_{i}^{N_{v}} {R\left( i \right)}^{2}\#\left( \text{1-1} \right) \end{aligned}$$

Entropy:

$$\begin{aligned} entropy=-\sum_{i=1}^{N_{r}} H\left( i \right)\log_{2} H\left( i \right)\#\left( \text{1-2} \right) \end{aligned}$$

Standard Entropy:

$$\begin{aligned} standard entropy=-\sum_{i=1}^{N_{r}} \left( \frac{H\left( i \right)}{N_{v}} \right)\log_{2} \left( \frac{H\left( i \right)}{N_{v}} \right)\#\left( \text{1}\text{-3} \right) \end{aligned}$$

Kurtosis:

$$\begin{aligned} kurtosis=\frac{\frac{1}{N_{v}}\sum_{i=1}^{N_{v}} \left( R\left( i \right)-\bar{R} \right)^{4}}{\left( \sqrt{\frac{1}{N_{v}}\sum_{i=1}^{N_{v}} \left( R\left( i \right)-\bar{R} \right)^{2}} \right)^{2}}\#\left( \text{1}\text{-4} \right) \end{aligned}$$

Mean:

$$\begin{aligned} mean=\frac{1}{N_{v}}\sum_{i}^{N_{v}} R\left( i \right)\#\left( \text{1}\text{-5} \right) \end{aligned}$$

Maximum: The maximum value of voxel in matrix R

Median: Median of voxel value in matrix R

Minimum: The minimum value of voxel in matrix R

Mass: Sum of voxel values in matrix R

Range: The range of voxel values in matrix R

Mean Absolute Deviation: Mean absolute deviation of voxel values in matrix R

Root Mean Square, RMS:

$$\begin{aligned} RMS=\sqrt{\frac{\sum_{i}^{N_{v}} {R\left( i \right)}^{2}}{N_{v}}}\#\left( \text{1}\text{-6} \right) \end{aligned}$$

Skewness:

$$\begin{aligned} skewness=\frac{\frac{1}{N_{v}}\sum_{i=1}^{N_{v}} \left( R\left( i \right)-\bar{R} \right)^{3}}{\left( \sqrt{\frac{1}{N_{v}}\sum_{i=1}^{N_{v}} \left( R\left( i \right)-\bar{R} \right)^{2}} \right)^{3}}\#\left( \text{1}\text{-7} \right) \end{aligned}$$

Standard Deviation:

$$\begin{aligned} standard deviation=\left( \frac{1}{N_{v}-1}\sum_{i=1}^{N_{v}} \left( R\left( i \right)-\bar{R} \right)^{2} \right)^{1/2}\#\left( \text{1}\text{-8} \right) \end{aligned}$$

Uniformity:

$$\begin{aligned} uniformity=\sum_{i=1}^{N_{r}} {H\left( i \right)}^{2}\#\left( \text{1}\text{-9} \right) \end{aligned}$$

Standard Uniformity:

$$\begin{aligned} standard uniformity=\sum_{i=1}^{N_{r}} \left( \frac{H\left( i \right)}{N_{v}} \right)^{2}\#\left( \text{1}\text{-10} \right) \end{aligned}$$

Variance:

$$\begin{aligned} variance=\frac{1}{N_{v}-1}\sum_{i=1}^{N_{v}} \left( R\left( i \right)-\bar{R} \right)^{2}\#\left( \text{1}\text{-11} \right) \end{aligned}$$

### Second order characteristic

Second order shape features reflect the volume, surface area and shape of brain regions, but they can not express the heterogeneity of brain regions. Shape features are similar to the semantic features commonly used in imaging. $V$: Volume of brain area (Volume), $A$: Surface Area.

Compactness 1:

$$\begin{aligned} compactness 1=\frac{V}{\sqrt{\pi}A^{\frac{2}{3}}}\#\left( \text{1}\text{-12} \right) \end{aligned}$$

Compactness 2:

$$\begin{aligned} compactness 2=36\pi\frac{V^{2}}{A^{3}}\#\left( \text{1}\text{-13} \right) \end{aligned}$$

Maximum 3D Diameter: The maximum Euclidean distance between voxels in ROI

Spherical Disproportion:

$$\begin{aligned} spherical disproportion=\frac{A}{4\pi R^{2}}\#\left( \text{1}\text{-14} \right) \end{aligned}$$

$R$: It's a sphere of the same size as the brain.

Sphericity:

$$\begin{aligned} sphericity=\frac{\pi^{\frac{1}{3}}\left( 6V \right)^{\frac{2}{3}}}{A}\#\left( \text{1}\text{-15} \right) \end{aligned}$$

Surface Area:

$$\begin{aligned} A=\sum_{i=1}^{N} \frac{1}{2}\left| a_{i}b_{i}\times a_{i}c_{i} \right|\#\left( \text{1}\text{-16} \right) \end{aligned}$$

$N$: It's the total number of triangles, $a$, $b$, $c$: It's the vector side of a triangle.

Surface to Volume Ratio:

$$\begin{aligned} surface to volume ratio=\frac{A}{V}\#\left( \text{1}\text{-17} \right) \end{aligned}$$

Volume: The volume of brain area is obtained by multiplying the number of ROI pixels by voxel size.

Second order texture features provide the information of image gray distribution, which can express the heterogeneity of brain regions. It is the most commonly used method in medical image analysis. The design of this study is extraction (Gray-Level Co-occurrence Matrix, GLCM), (Gray-Level Run-length Matrix, GLRLM, (Gray-Level Size Zone Matrix, GLSZM), (Neighborhood Gray-Tone Difference Matrix, NGTDM). There are 51 texture features. In this study, the voxel intensity value in ROI is discretized, the gray level is defined as 25, the image is resampled, and then the following texture features are extracted.

#### Gray-Level Co-occurrence Matrix, GLCM

Gray level co-occurrence matrix can reflect the arrangement of voxels. Let R (i, j; δ, α) be the gray level co-occurrence matrix, (I, J) be the combination times of intensity values i and j of two "co-occurrence" voxels; δ: It is the distance between two "symbiotic" voxels, which is set as 1 in this study; α: In 3-D matrix, a voxel has 26 adjacent voxels, that is, 13 directions. Let R (I, J) be α co-occurrence matrix of direction, μ is the mean value of R (I, J). Let n_ R be the number of discrete gray levels contained in the ROI matrix, and the size of GLCM is N_r × N_r.

Edge probability of row:

$$\begin{aligned} {Ep}_{x}\left( i \right)=\sum_{j=1}^{N_{r}} R\left( i,j \right)\#\left( \text{1}\text{-18} \right) \end{aligned}$$

Suppose the edge probability of a column:

$$\begin{aligned} {Ep}_{y}\left( i \right)=\sum_{i=1}^{N_{r}} R\left( i,j \right)\#\left( \text{1}\text{-19} \right) \end{aligned}$$

$\mu_{x}$: the mean of ${Ep}_{x}$; $\mu_{y}$: the mean of ${Ep}_{y}$; $\sigma_{x}$: The standard deviation of ${Ep}_{x}$; $\sigma_{y}: The standard deviation of{Ep}_{y}$.

$$\begin{aligned} {Ep}_{x+y}\left( k \right)=\sum_{i=1}^{N_{r}} \sum_{j=1}^{N_{r}} R\left( i,j \right), i+j=k, k=2,3,\ldots,2N_{r}\#\left( \text{1}\text{-20} \right) \end{aligned}$$

$$\begin{aligned} {Ep}_{x-y}\left( k \right)=\sum_{i=1}^{N_{r}} \sum_{j=1}^{N_{r}} R\left( i,j \right), \left| i-j \right|=k,k=0,1,\ldots,N_{r}-1\#\left( \text{1}\text{-21} \right) \end{aligned}$$

$$\begin{aligned} {EN}_{x}=-\sum_{i=1}^{N_{r}} {Ep}_{x}\left( i \right)\log_{2} \left[ {Ep}_{x}\left( i \right) \right]\#\left( \text{1}\text{-22} \right) \end{aligned}$$

$$\begin{aligned} {EN}_{y}=-\sum_{i=1}^{N_{r}} {Ep}_{y}\left( i \right)\log_{2} \left[ {Ep}_{y}\left( i \right) \right]\#\left( \text{1}\text{-23} \right) \end{aligned}$$

$$\begin{aligned} {EN}_{R}=-\sum_{i=1}^{N_{r}} \sum_{j=1}^{N_{r}} R\left( i,j \right)\log_{2} \left[ R\left( i,j \right) \right]\#\left( \text{1}\text{-24} \right) \end{aligned}$$

$$\begin{aligned} {EN}_{XY1}=-\sum_{i=1}^{N_{r}} \sum_{j=1}^{N_{r}} R\left( i,j \right)\log\left( {Ep}_{x}\left( i \right){Ep}_{y}\left( j \right) \right)\#\left( \text{1}\text{-25} \right) \end{aligned}$$

$$\begin{aligned} {EN}_{XY2}=-\sum_{i=1}^{N_{r}} \sum_{j=1}^{N_{r}} {Ep}_{x}\left( i \right){Ep}_{y}\left( j \right)\log\left( {Ep}_{x}\left( i \right){Ep}_{y}\left( j \right) \right)\#\left( \text{1}\text{-26} \right) \end{aligned}$$

Autocorrelation:

$$\begin{aligned} autocorrelation=\sum_{i=1}^{N_{r}} \sum_{j=1}^{N_{r}} ijR\left( i,j \right)\#\left( \text{1}\text{-27} \right) \end{aligned}$$

Cluster Prominence:

$$\begin{aligned} cluster prominence=\sum_{i=1}^{N_{r}} \sum_{j=1}^{N_{r}} \left[ i+j-\mu_{x}\left( i \right)-\mu_{y}\left( j \right) \right]^{4}R\left( i,j \right)\#\left( \text{1}\text{-28} \right) \end{aligned}$$

Cluster Shade:

$$\begin{aligned} cluster shade=\sum_{i=1}^{N_{r}} \sum_{j=1}^{N_{r}} \left[ i+j-\mu_{x}\left( i \right)-\mu_{y}\left( j \right) \right]^{3}R\left( i,j \right)\#\left( \text{1}\text{-29} \right) \end{aligned}$$

Cluster Tendency:

$$\begin{aligned} cluster tendency=\sum_{i=1}^{N_{r}} \sum_{j=1}^{N_{r}} \left[ i+j-\mu_{x}\left( i \right)-\mu_{y}\left( j \right) \right]^{2}R\left( i,j \right)\#\left( \text{1}\text{-30} \right) \end{aligned}$$

Contrast:

$$\begin{aligned} contrast=\sum_{i=1}^{N_{r}} \sum_{j=1}^{N_{r}} \left| i-j \right|^{2}R\left( i,j \right)\#\left( \text{1}\text{-31} \right) \end{aligned}$$

Correlation:

$$\begin{aligned} correlation=\frac{\sum_{i=1}^{N_{r}} \sum_{j=1}^{N_{r}} ijR\left( i,j \right)-\mu_{i}{\left( i \right)\mu}_{j}\left( j \right)}{\sigma_{x}\left( i \right)\sigma_{y}\left( j \right)}\#\left( \text{1-32} \right) \end{aligned}$$

Difference Entropy:

$$\begin{aligned} difference entropy=\sum_{i=0}^{N_{r}-1} R_{x-y}\left( i \right)\log_{2} \left[ R_{x-y}\left( i \right) \right]\#\left( 1\text{-33} \right) \end{aligned}$$

Dissimilarity:

$$\begin{aligned} dissimilarity=\sum_{i=1}^{N_{r}} \sum_{j=1}^{N_{r}} \left| i-j \right|R\left( i,j \right)\#\left( 1\text{-34} \right) \end{aligned}$$

Energy:

$$\begin{aligned} energy=\sum_{i=1}^{N_{r}} \sum_{j=1}^{N_{r}} \left[ R\left( i,j \right) \right]^{2}\#\left( \text{1-35} \right) \end{aligned}$$

Entropy ($H$):

$$\begin{aligned} entropy=-\sum_{i=1}^{N_{r}} \sum_{j=1}^{N_{r}} R\left( i,j \right)\log_{2} \left[ R\left( i,j \right) \right]\#\left( 1\text{-36} \right) \end{aligned}$$

Homogeneity 1:

$$\begin{aligned} homogeneity 1=\sum_{i=1}^{N_{r}} \sum_{j=1}^{N_{r}} \frac{R\left( i,j \right)}{1+\left| i-j \right|}\#\left( \text{1-37} \right) \end{aligned}$$

Homogeneity 2:

$$\begin{aligned} homogeneity 2=\sum_{i=1}^{N_{r}} \sum_{j=1}^{N_{r}} \frac{R\left( i,j \right)}{1+\left| i-j \right|^{2}}\#\left( \text{1}\text{-38} \right) \end{aligned}$$

Informational Measure of Correlation, ICMC 1 (IMC1):

$$\begin{aligned} IMC1=\frac{{EN}_{R}-{EN}_{XY1}}{\text{max}\left\{ {EN}_{x},{EN}_{y} \right\}}\#\left( \text{1-39} \right) \end{aligned}$$

Informational Measure of Correlation, ICMC 2 (IMC2):

$$\begin{aligned} IMC2=\sqrt{1-e^{-2\left( {EN}_{XY2}-{EN}_{R} \right)}}\#\left( \text{1}\text{-40} \right) \end{aligned}$$

Inverse Difference Moment Normalized , IDMN:

$$\begin{aligned} IDMN=\sum_{i=1}^{N_{r}} \sum_{j=1}^{N_{r}} \frac{R\left( i,j \right)}{1+\left( \frac{\left| i-j \right|^{2}}{{N_{r}}^{2}} \right)}\#\left( \text{1-41} \right) \end{aligned}$$

Inverse Difference Normalized, IDN:

$$\begin{aligned} IDN=\sum_{i=1}^{N_{r}} \sum_{j=1}^{N_{r}} \frac{R\left( i,j \right)}{1+\left( \frac{\left| i-j \right|}{N_{r}} \right)}\#\left( 1\text{-42} \right) \end{aligned}$$

Inverse Variance:

$$\begin{aligned} \begin{matrix} inverse variance=\sum_{i=1}^{N_{r}} \sum_{j=1}^{N_{r}} \frac{R\left( i,j \right)}{\left| i-j \right|^{2}} & ,i\neq j \end{matrix}\#\left( \text{1}\text{-43} \right) \end{aligned}$$

Maximum Probability:

$$\begin{aligned} maximum probability=\text{max}\left\{ R\left( i,j \right) \right\}\#\left( \text{1}\text{-44} \right) \end{aligned}$$

Sum Average:

$$\begin{aligned} sum average=\sum_{i=2}^{2N_{r}} \left[ iR_{x+y}\left( i \right) \right]\#\left( \text{1}\text{-45} \right) \end{aligned}$$

Sum Entropy:

$$\begin{aligned} sum entropy=-\sum_{i=2}^{2N_{r}} R_{x+y}\left( i \right)\log_{2} \left[ R_{x+y}\left( i \right) \right]\#\left( \text{1}\text{-46} \right) \end{aligned}$$

Sum Variance:

$$\begin{aligned} sum variance=\sum_{i=2}^{2N_{r}} \left( i-SE \right)^{2}R_{x+y}\left( i \right)\#\left( \text{1}\text{-47} \right) \end{aligned}$$

Variance:

$$\begin{aligned} variance=\sum_{i=1}^{N_{r}} \sum_{j=1}^{N_{r}} \left( i-\mu\right)^{2}R\left( i,j \right)\#\left( \text{1}\text{-48} \right) \end{aligned}$$

#### Gray-Level Run-length Matrix, GLRLM

Short Run Emphasis (SRE):

$$\begin{aligned} SRE=\frac{\sum_{i=1}^{N_{r}} \sum_{j=1}^{N_{l}} \left[ \frac{R\left( i,j | \theta\right)}{j^{2}} \right]}{\sum_{i=1}^{N_{r}} \sum_{j=1}^{N_{l}} R\left( i,j | \theta\right)}.\#\left( \text{1}\text{-49} \right) \end{aligned}$$

Long Run Emphasis (LRE):

$$\begin{aligned} LRE=\frac{\sum_{i=1}^{N_{r}} \sum_{j=1}^{N_{l}} j^{2}R\left( i,j | \theta\right)}{\sum_{i=1}^{N_{r}} \sum_{j=1}^{N_{l}} R\left( i,j | \theta\right)}.\#\left( \text{1}\text{-50} \right) \end{aligned}$$

Gray Level Non-Uniformity (GLN):

$$\begin{aligned} GLN=\frac{\sum_{i=1}^{N_{r}} \left[ \sum_{j=1}^{N_{l}} R\left( i,j | \theta\right) \right]^{2}}{\sum_{i=1}^{N_{r}} \sum_{j=1}^{N_{l}} R\left( i,j | \theta\right)}.\#\left( \text{1}\text{-51} \right) \end{aligned}$$

Run Length Non-Uniformity (RLN):

$$\begin{aligned} RLN=\frac{\sum_{j=1}^{N_{l}} \left[ \sum_{i=1}^{N_{r}} R\left( i,j | \theta\right) \right]^{2}}{\sum_{i=1}^{N_{r}} \sum_{j=1}^{N_{l}} R\left( i,j | \theta\right)}.\#\left( \text{1}\text{-52} \right) \end{aligned}$$

Run Percentage (RP):

$$\begin{aligned} RP=\sum_{i=1}^{N_{r}} \sum_{j=1}^{N_{l}} \frac{R\left( i,j | \theta\right)}{N_{v}}.\#\left( \text{1}\text{-53} \right) \end{aligned}$$

Low Gray Level Run Emphasis (LGLRE):

$$\begin{aligned} LGLRE=\frac{\sum_{i=1}^{N_{r}} \sum_{j=1}^{N_{l}} \left[ \frac{R\left( i,j | \theta\right)}{i^{2}} \right]}{\sum_{i=1}^{N_{r}} \sum_{j=1}^{N_{l}} R\left( i,j | \theta\right)}.\#\left( \text{1}\text{-54} \right) \end{aligned}$$

High Gray Level Run Emphasis (HGLRE):

$$\begin{aligned} HGLRE=\frac{\sum_{i=1}^{N_{r}} \sum_{j=1}^{N_{l}} i^{2}R\left( i,j | \theta\right)}{\sum_{i=1}^{N_{r}} \sum_{j=1}^{N_{l}} R\left( i,j | \theta\right)}.\#\left( \text{1}\text{-55} \right) \end{aligned}$$

Short Run Low Gray Level Emphasis (SRLGLE):

$$\begin{aligned} SRLGLE=\frac{\sum_{i=1}^{N_{r}} \sum_{j=1}^{N_{l}} \left[ \frac{R\left( i,j | \theta\right)}{i^{2}j^{2}} \right]}{\sum_{i=1}^{N_{r}} \sum_{j=1}^{N_{l}} R\left( i,j | \theta\right)}.\#\left( \text{1}\text{-56} \right) \end{aligned}$$

Short Run High Gray Level Emphasis (SRHGLE):

$$\begin{aligned} SRHGLE=\frac{\sum_{i=1}^{N_{r}} \sum_{j=1}^{N_{l}} \left[ \frac{R\left( i,j | \theta\right)i^{2}}{j^{2}} \right]}{\sum_{i=1}^{N_{r}} \sum_{j=1}^{N_{l}} R\left( i,j | \theta\right)}.\#\left( \text{1}\text{-57} \right) \end{aligned}$$

Long Run Low Gray Level Emphasis (LRLGLE):

$$\begin{aligned} LRLGLE=\frac{\sum_{i=1}^{N_{r}} \sum_{j=1}^{N_{l}} \left[ \frac{R\left( i,j | \theta\right)j^{2}}{i^{2}} \right]}{\sum_{i=1}^{N_{r}} \sum_{j=1}^{N_{l}} R\left( i,j | \theta\right)}.\#\left( \text{1}\text{-58} \right) \end{aligned}$$

Long Run High Gray Level Emphasis (LRHGLE):

$$\begin{aligned} LRHGLE=\frac{\sum_{i=1}^{N_{r}} \sum_{j=1}^{N_{l}} R\left( i,j | \theta\right)i^{2}j^{2}}{\sum_{i=1}^{N_{r}} \sum_{j=1}^{N_{l}} R\left( i,j | \theta\right)}.\#\left( \text{1}\text{-59} \right) \end{aligned}$$

#### Gray-Level Size Zone Matrix, GLSZM

$$\begin{aligned} r\left( i,j \right)=\frac{R\left( i,j \right)}{\sum_{i=1}^{N_{r}} \sum_{j=1}^{L_{z}} R\left( i,j \right)}.\#\left( \text{1}\text{-60} \right) \end{aligned}$$

$$\begin{aligned} \mu_{i}=\sum_{i=1}^{N_{r}} i\sum_{j=1}^{L_{z}} r\left( i,j \right), \mu_{j}=\sum_{j=1}^{L_{z}} j\sum_{i=1}^{N_{r}} r\left( i,j \right).\#\left( \text{1}\text{-61} \right) \end{aligned}$$

Short Zone Emphasis (SZE):

$$\begin{aligned} SZE=\sum_{i=1}^{N_{r}} \sum_{j=1}^{L_{z}} \left[ \frac{r\left( i,j \right)}{j^{2}} \right].\#\left( \text{1}\text{-62} \right) \end{aligned}$$

Large Zone Emphasis (LZE):

$$\begin{aligned} LZE=\sum_{i=1}^{N_{r}} \sum_{j=1}^{L_{z}} j^{2}r\left( i,j \right).\#\left( \text{1}\text{-63} \right) \end{aligned}$$

Gray Level Non-uniformity (GLN):

$$\begin{aligned} GLN=\sum_{i=1}^{N_{r}} \left[ \sum_{j=1}^{L_{z}} r\left( i,j \right) \right]^{2}.\#\left( \text{1}\text{-64} \right) \end{aligned}$$

Zone Size Non-uniformity (ZSN):

$$\begin{aligned} ZSN=\sum_{j=1}^{L_{z}} \left[ \sum_{i=1}^{N_{r}} r\left( i,j \right) \right]^{2}.\#\left( \text{1}\text{-65} \right) \end{aligned}$$

Zone Percentage (ZP):

$$\begin{aligned} ZP=\frac{\sum_{i=1}^{N_{r}} \sum_{j=1}^{L_{z}} r\left( i,j \right)}{\sum_{j=1}^{L_{z}} j\sum_{i=1}^{N_{r}} r\left( i,j \right)}.\#\left( \text{1}\text{-66} \right) \end{aligned}$$

Low Gray-level Zone Emphasis (LGZE):

$$\begin{aligned} LGZE=\sum_{i=1}^{N_{r}} \sum_{j=1}^{L_{z}} \left[ \frac{r\left( i,j \right)}{i^{2}} \right].\#\left( \text{1-67} \right) \end{aligned}$$

High Gray-level Zone Emphasis (HGZE):

$$\begin{aligned} HGZE=\sum_{i=1}^{N_{r}} \sum_{j=1}^{L_{z}} i^{2}r\left( i,j \right).\#\left( \text{1}\text{-68} \right) \end{aligned}$$

Small Zone Low Gray-level Emphasis (SZLGE):

$$\begin{aligned} SZLGE=\sum_{i=1}^{N_{r}} \sum_{j=1}^{L_{z}} \left[ \frac{r\left( i,j \right)}{i^{2}j^{2}} \right].\#\left( \text{1}\text{-69} \right) \end{aligned}$$

Small Zone High Gray-level Emphasis (SZHGE):

$$\begin{aligned} SZHGE=\sum_{i=1}^{N_{r}} \sum_{j=1}^{L_{z}} \left[ \frac{i^{2}r\left( i,j \right)}{j^{2}} \right].\#\left( \text{1}\text{-70} \right) \end{aligned}$$

Large Zone Low Gray-level Emphasis (LZLGE):

$$\begin{aligned} LZLGE=\sum_{i=1}^{N_{r}} \sum_{j=1}^{L_{z}} \left[ \frac{j^{2}r\left( i,j \right)}{i^{2}} \right].\#\left( \text{1-71} \right) \end{aligned}$$

Large Zone High Gray-level Emphasis (LZHGE):

$$\begin{aligned} LZHGE=\sum_{i=1}^{N_{r}} \sum_{j=1}^{L_{z}} i^{2}j^{2}r\left( i,j \right).\#\left( 1\text{-72} \right) \end{aligned}$$

Gray-Level Variance (GLV):

$$\begin{aligned} GLV=\frac{1}{N_{r}\times L_{z}}\sum_{i=1}^{N_{r}} \sum_{j=1}^{L_{z}} \left( ir\left( i,j \right)-\mu_{i} \right)^{2}.\#\left( 1\text{-73} \right) \end{aligned}$$

Zone-Size Variance (ZSV):

$$\begin{aligned} ZSV=\frac{1}{N_{r}\times L_{z}}\sum_{i=1}^{N_{r}} \sum_{j=1}^{L_{z}} \left( jr\left( i,j \right)-\mu_{j} \right)^{2}.\#\left( \text{1}\text{-74} \right) \end{aligned}$$

#### Neighborhood Gray-Tone Difference Matrix, NGTDM

$$\begin{aligned} P\left( i \right)=\left\{ \begin{aligned} \sum_{all voxels \in\left\{ N_{i} \right\}} \left| i-\bar{A_{i}} \right| if N_{i}>0, \\ 0 if N_{i}=0 \end{aligned} \right.\#\left( \text{1-75} \right) \end{aligned}$$

$$\begin{aligned} \bar{A}_{i}=\bar{A}\left( j,k,l \right)=\frac{\sum_{m=-1}^{m=1} \sum_{n=-1}^{n=1} \sum_{o=-1}^{o=1} \omega_{m,n,o}\cdot V\left( j+m,k+n,l+o \right)}{\sum_{m=-1}^{m=1} \sum_{n=-1}^{n=1} \sum_{o=-1}^{o=1} \omega_{m,n,o}}\#\left( \text{1-76} \right) \end{aligned}$$

$$\begin{aligned} \omega_{m,n,o}=\left\{ \begin{aligned} 1 if \left| j-m \right|+\left| k-n \right|+\left| l-o \right|=1 \\ \frac{1}{\sqrt{2}} if \left| j-m \right|+\left| k-n \right|+\left| l-o \right|=2 \\ \frac{1}{\sqrt{3}} if \left| j-m \right|+\left| k-n \right|+\left| l-o \right|=3 \\ 0 if V\left( j+m,k+n,l+o \right) is undefined \end{aligned} \right.\#\left( 1\text{-77} \right) \end{aligned}$$

$$\begin{aligned} n_{i}=\frac{N_{i}}{N}\#\left( 1\text{-78} \right) \end{aligned}$$

Coarseness:

$$\begin{aligned} coarseness=\left[ \varepsilon+\sum_{i=1}^{N_{g}} n_{i}P\left( i \right) \right]^{-1}\#\left( \text{1-79} \right) \end{aligned}$$

Among ε it is a normal number to prevent coarseness from producing infinite results $1\times{10}^{-6}$.

Contrast:

$$\begin{aligned} contrast=\left[ \frac{1}{\left( N_{g} \right)_{eff}\left[ \left( N_{g} \right)_{eff}-1 \right]}\sum_{i=1}^{N_{g}} \sum_{j=1}^{N_{g}} n_{i}n_{j}\left( i-j \right)^{2} \right]\left[ \frac{1}{N}\sum_{i=1}^{N_{g}} P\left( i \right) \right].\#\left( 1\text{-80} \right) \end{aligned}$$

Busyness:

$$\begin{aligned} busyness=\frac{\sum_{i=1}^{N_{g}} n_{i}P\left( i \right)}{\sum_{i=1}^{N_{g}} \sum_{j=1}^{N_{g}} \left( in_{i}-jn_{j} \right)}, n_{i}\neq0, n_{j}\neq0.\#\left( 1\text{-81} \right) \end{aligned}$$

Complexity:

$$\begin{aligned} complexity=\sum_{i=1}^{N_{g}} \sum_{j=1}^{N_{g}} \frac{\left| i-j \right|\left[ \left( iP\left( i \right)+P\left( j \right)n_{j} \right) \right]}{N\left( n_{i}-n_{j} \right)}, n_{i}\neq0, n_{j}\neq0.\#\left( \text{1}\text{-82} \right) \end{aligned}$$

Strength:

$$\begin{aligned} strength=\frac{\sum_{i=1}^{N_{g}} \sum_{j=1}^{N_{g}} \left( n_{i}+n_{j} \right)\left( i-j \right)^{2}}{\left[ \varepsilon+\sum_{i=1}^{N_{g}} P\left( i \right) \right]}, n_{i}\neq0, n_{j}\neq0.\#\left( \text{1}\text{-83} \right) \end{aligned}$$

Among ε it is a normal number to prevent coarseness from producing infinite results $1\times{10}^{-6}$.

### Wavelet features

The wavelet features in this study are obtained by calculating the first-order features and the second-order features through the "coiflet 1" 3-D wavelet transform of brain area (ROI). Let l be a low pass, h be a high pass, and the original matrix be X: $X_{LLL}$, $X_{LLH}$,$X_{LHL}$,$X_{LHH}$,$X_{HLL}$,$X_{HLH}$, $X_{HHL}$, $X_{HHH}$.
